# IL-36/LXR axis modulates cholesterol metabolism and immune defense to *Mycobacterium tuberculosis*

**DOI:** 10.1038/s41598-018-19476-x

**Published:** 2018-01-24

**Authors:** Fadhil Ahsan, Jeroen Maertzdorf, Ute Guhlich-Bornhof, Stefan H. E. Kaufmann, Pedro Moura-Alves

**Affiliations:** Department of Immunology, Max Planck Institute for Infection Biology, Charitéplatz 1, Berlin, 10117 Germany

## Abstract

*Mycobacterium tuberculosis* (*Mtb*) is a life-threatening pathogen in humans. Bacterial infection of macrophages usually triggers strong innate immune mechanisms, including IL-1 cytokine secretion. The newer member of the IL-1 family, IL-36, was recently shown to be involved in cellular defense against *Mtb*. To unveil the underlying mechanism of IL-36 induced antibacterial activity, we analyzed its role in the regulation of cholesterol metabolism, together with the involvement of Liver X Receptor (LXR) in this process. We report that, in *Mtb*-infected macrophages, IL-36 signaling modulates cholesterol biosynthesis and efflux via LXR. Moreover, IL-36 induces the expression of cholesterol-converting enzymes and the accumulation of LXR ligands, such as oxysterols. Ultimately, both IL-36 and LXR signaling play a role in the regulation of antimicrobial peptides expression and in *Mtb* growth restriction. These data provide novel evidence for the importance of IL-36 and cholesterol metabolism mediated by LXR in cellular host defense against *Mtb*.

## Introduction

*Mycobacterium tuberculosis* (*Mtb*) is an intracellular pathogen that caused 1.7 million human casualties in 2016^1^. The bacterium preferentially resides and replicates in macrophages^2^, which are key components of innate host defense and play a central role in tuberculosis pathology^3^. At the frontline of host defense, macrophages also are the main producers of cytokines mediating inflammatory responses and antimicrobial effector mechanisms^4,5^. Amongst the pro-inflammatory cytokines produced by macrophages upon *Mtb* infection are the IL-36 cognates of the IL-1 family^6–8^. These comprise the molecules IL-36α, IL-36β, IL-36γ and the putative IL-36 receptor antagonist (IL-36Ra), which specifically binds to the IL-36 receptor (IL-36R)^9,10^. Amongst these, IL-36γ is most profoundly induced upon *Mtb* infection^7,8^. Several studies have recognized the role of these cytokines in host defense against *Mtb* and other bacteria, by regulating the expression of antimicrobial peptides (APs), such as cathelicidin and defensins^7,11,12^.

Since host cholesterol is a crucial carbon source for persistent *Mtb* infection^13^, modulating the cholesterol metabolism could be a potential strategy to control *Mtb*. Liver X Receptor (LXR), a master regulator of cholesterol, contributes to host resistance against tuberculosis in mice and human^14,15^. LXR consists of two sterol-responsive nuclear receptors, LXRα and LXRβ, which act as transcription factors and bind to LXR element (LXRE) sequences in the promoters of several genes^16^. Amongst these are several genes encoding proteins involved in cellular cholesterol turnover such as ATP-binding cassette transporter A1 (ABCA1), ATP-binding cassette transporter G1 (ABCG1), Apolipoprotein E (APOE) and inducible degrader of low density lipoprotein receptor (IDOL)^16,17^. ABCA1 and ABCG1 are membrane transporters, APOE is a potent cholesterol acceptor mediating cholesterol efflux^16^, and IDOL can limit cholesterol uptake by reducing LDLR expression^17^. Furthermore, a recent study revealed a role of LXR in suppressing cholesterol biosynthesis^18^. Activation of LXR is driven by various synthetic and naturally occurring-endogenous ligands such as oxysterols^16,19^. Although LXR activation upon *Mtb* infection has been reported^14^, little is known about the endogenous factors driving this activation.

Here we report a novel link between IL-36 signaling and cholesterol metabolism. We demonstrate first that upon *Mtb* infection, IL-36 regulates cholesterol synthesis through the induction of LXR. Second, we find that IL-36 activity is involved in the regulation of oxysterols and production of AP that control *Mtb* growth. We conclude that coordinated IL-36 and LXR signaling plays a crucial role in host defense against *Mtb*.

## Results

### IL-36 signaling facilitates LXR activation upon *Mtb* infection

**Figure 1:**
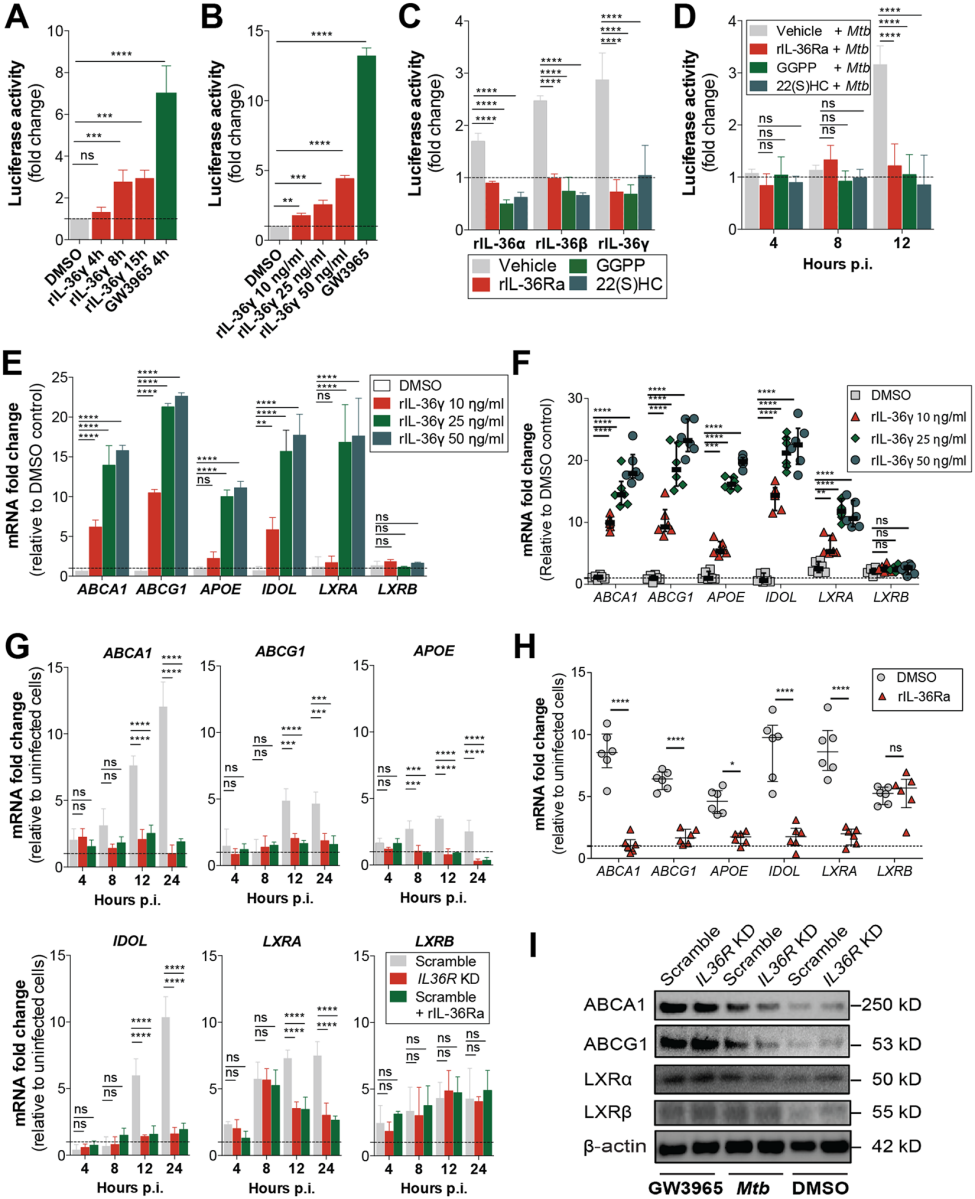
IL-36 signaling is required for LXR activation upon *Mtb* infection in human macrophages. (**A**–**D**) LXR luciferase reporter activity in THP-1 macrophages stimulated with (**A**) rIL-36γ (25 ng/ml), (**B**) increasing concentrations of rIL-36γ at 8 h, (**C**) all IL-36 variants (at 25 ng/ml for 8 h) and (**D**) *Mtb* infection at the specified time points after pre-incubation with vehicle, rIL-36Ra (100 ng/ml, 3 h), GGPP (25 μM, 15 h) and 22(S)HC (10 μM, 3 h). (**E**,**F**,**G** and **H**) Induction of gene expression of LXR target genes and receptors in THP-1 macrophages (**E**) and MDMs (**F**) stimulated with rIL-36γ for 8 h and upon *Mtb* infection with or without blocking IL-36 signaling (**G** and **H**). (**I**) Immunoblot of ABCA1, ABCG1, LXRα and LXRβ protein levels from *Mtb*-infected scramble and *IL36R* KD macrophages at 24 h p.i. GW3965 (500 nM) was used as positive control. (**A**–**E**,**G**) Data pooled from three independent experiments are shown. Data are shown as mean ± SD. (**F** and **H**) Data from one representative experiment out of three independent experiments are shown. Data are shown as median ± interquartile range, with each dot of MDM representing one human donor. (**I**) Data from one representative experiment of two independent experiments are shown. *P* values shown as ns p > 0.05; *p ≤ 0.05; **p ≤ 0.01; ***p ≤ 0.001; ****p ≤ 0.0001.

Following up on our previous work on IL-36 induction upon *Mtb* infection and its antibacterial effect in macrophages^7^, we aimed to get a broader view of the IL-36 dependent signaling pathways involved in the control of infection. For this, we generated gene expression profiles from infected control (scramble) and IL-36R knockdown cells and analyzed the differentially expressed gene profiles. Ingenuity Pathway analysis (IPA) revealed a clear enrichment of genes involved in cholesterol metabolism whereby most genes were higher expressed in the IL-36R deficient cells (Supplementary Figure 1A). Since cholesterol biosynthesis can be directly regulated by LXR^18^, we decided to evaluate whether IL-36 is able to regulate cholesterol metabolism via this pathway. To this end, we generated a THP-1 LXR luciferase macrophage reporter cell line. LXR specific activation was confirmed using GW3965, a specific LXR synthetic ligand, in the presence or absence of LXR inhibitors GGPP and 22(S)HC (Supplementary Figure 1B)^20,21^. LXR reporter macrophages were then stimulated with recombinant IL-36γ (rIL-36γ), resulting in activation of LXR in a dose dependent manner (Fig. 1A,B). LXR activation was also induced by the other IL-36 cognates, rIL-36α and rIL-36β, which could be blocked by recombinant IL-36 receptor antagonist (rIL-36Ra) or by LXR inhibitors (Fig. 1C). At the concentrations tested, rIL-36Ra and GGPP did not affect cell viability (Supplementary Figure 1C).

Considering that *Mtb* infection triggers the secretion of IL-36^7^, we evaluated whether LXR activity was altered upon infection. Similar to IL-36 stimulation, infection with *Mtb* significantly induced LXR activation, which could be blocked by rIL-36Ra or LXR inhibitors (Fig. 1D). To further assess LXR activation by IL-36 stimulation and *Mtb* infection, we measured the expression of LXR target genes *ABCA1*, *ABCG1*, *APOE* and *IDOL*, as well as the LXR receptors (*LXRA* and *LXRB*)^16^ in THP-1 macrophages and in primary monocyte-derived macrophages (MDM). Consistent with the THP-1 LXR reporter cell line, rIL-36γ stimulation induced the expression of LXR target genes and *LXRA* in THP-1 macrophages and MDM (Fig. 1E,F). The expression of *LXRB* was not altered, which is in agreement with previous studies showing that *LXRB* is not a direct target of LXR^22,23^. We also confirmed the role of IL-36 signaling in the activation of LXR upon *Mtb* infection, either by knocking down the IL-36 receptor (*IL36R*) or using the rIL-36Ra^7^, resulting in significantly lower induction of LXR downstream genes (Fig. 1G,H). Moreover, *Mtb* infection affected the protein levels of LXR induced genes in an IL-36 signaling dependent manner (Fig. 1I). These results suggest that *Mtb* infection activates the LXR pathway through IL-36.

### Recombinant IL-36γ facilitates the production of endogenous LXR ligands

**Figure 2:**
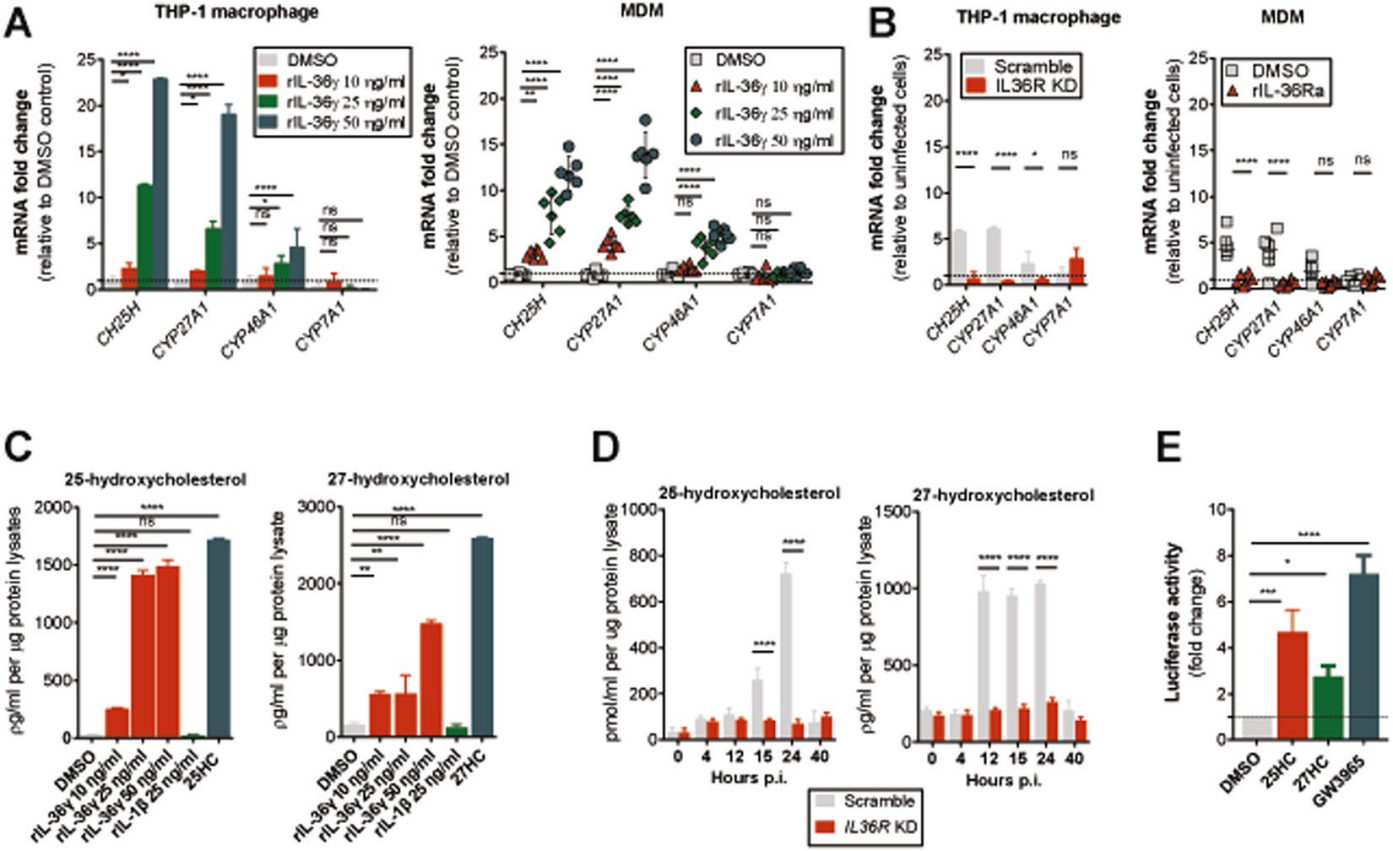
rIL-36γ drives expression of cholesterol-converting enzymes and endogenous LXR ligands. (**A** and **B**) mRNA expression of *CH25H*, *CYP27A1*, *CYP46A1* and *CYP7A1* in (**A**) THP-1 macrophages and MDM upon 4 h of stimulation with various doses of rIL-36γ and (**B**) 12 h *Mtb*-infection with or without blocking IL-36R signaling. (**C** and **D**) Concentrations of intracellular 25HC or 27HC in cell lysates of THP-1 macrophages (**C**) upon stimulation with rIL-36γ or rIL-1β or (**D**) after *Mtb* infection. (**E**) LXR luciferase activity in THP-1 macrophages treated with DMSO or 500 nM of 25HC, 27HC or GW3965 for 8 h. (**A–E**) Data from THP-1 macrophages are pooled from three independent experiments and shown as mean ± SD. MDM data are from one representative experiment of three independent experiments, median ± interquartile range is shown and each dot of MDM represents one donor. *P* values shown as ns *p* > 0.05; *p ≤ 0.05; **p ≤ 0.01; ***p ≤ 0.001; ****p ≤ 0.0001.

To further extend our knowledge on how IL-36 activates LXR, we assessed whether rIL-36γ can drive the production of endogenous LXR ligands. It has been reported that activation of LXR can be triggered by endogenous oxysterols^24–26^. Several cholesterol-converting enzymes encoded by the genes *CH25H*, *CYP27A1*, *CYP46A1* and *CYP7A1* can convert free cholesterol into oxysterols 25HC, 27HC, 24HC and 7αHC, respectively^27,28^. We found that *CH25H* and *CYP27A1* were strongly induced by rIL-36γ in both THP-1 macrophages and MDM (Fig. 2A). Strikingly, induction of *CH25H* and *CYP27A1* was also observed in *Mtb*-infected cells, in an IL-36 signaling dependent manner (Fig. 2B). We therefore evaluated whether IL-36 can also induce expression of 25HC and 27HC. Indeed, IL-36, but not IL-1β, stimulated the expression of both oxysterols (Fig. 2C), as did *Mtb* infection in IL-36 signaling competent cells (Fig. 2D). Finally, we showed that both 25HC and 27HC could directly activate LXR (Fig. 2E). These results point to a mechanism where, in response to *Mtb* infection, IL-36 induces the conversion of oxysterols leading to LXR activation.

### IL-36-induced host defense in macrophage is LXR-dependent

**Figure 3:**
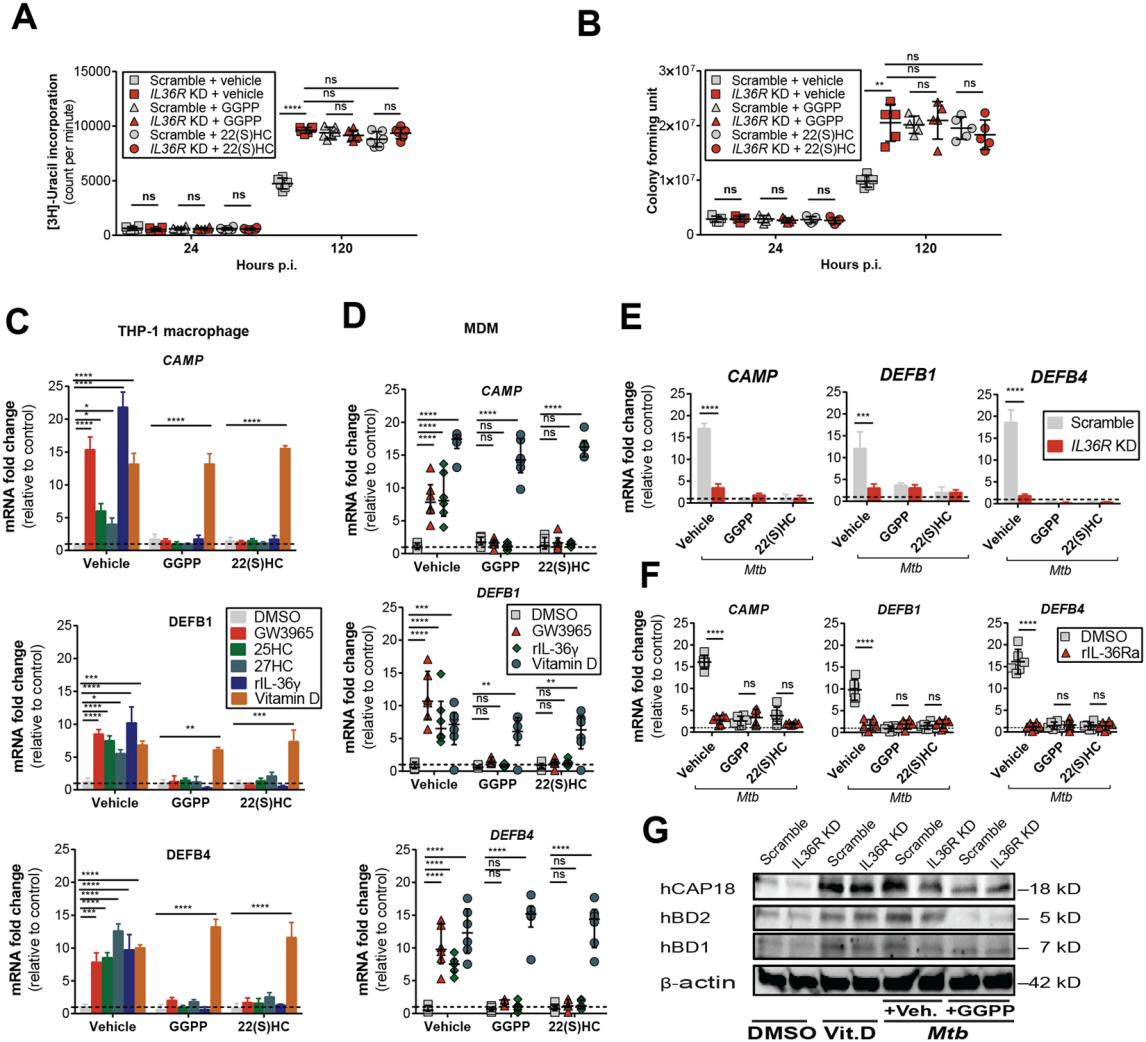
IL-36-induced LXR activation inhibits *Mtb* growth in macrophages. Bacterial growth in *IL36R* KD cells assessed by [3 H]-uracil uptake (**A**) and colony forming units (**B**) after pre-treatment with vehicle, GGPP or 22(S)HC. (**C** and **D**) APs mRNA expression in (**C**) THP-1 macrophages and (**D**) MDMs upon stimulation with the indicated compounds, (**E**) upon 24 h *Mtb* infection in IL-36 signaling blocked THP-1 macrophages and (**F**) MDM. (**G**) Intracellular protein levels of hCAP18 (cathelicidin), hBD2 (β-defensin 2) and hBD1 (β-defensin 1) upon 30 h *Mtb* infection of scramble or *IL36R* KD THP-1 macrophages with or without LXR inhibitor. Vitamin D and beta actin were used as positive control for AP induction and protein loading control, respectively. (**A**,**B**,**D** and **F**) Data from one representative experiment of at least three independent experiments are shown. Each dot of MDM represents one donor. MDM and bacterial counts are shown as median ± interquartile range. (**C** and **E**) Data pooled from three independent experiments are shown and represented as mean ± SD. (**G**) Data representative of one experiment of two independent experiments are shown. *P* values shown as ns *p* > 0.05; *p ≤ 0.05; **p ≤ 0.01; ***p ≤ 0.001; ****p ≤ 0.0001.

As previously reported^7^, IL-36 signaling plays an important role in restricting bacterial growth. Surprisingly, we found that blocking LXR activation led to a similar increase in bacterial growth, as in the absence of IL-36 signaling (Fig. 3A,B). Of note, we found a significant impact of 22(S)HC on cell viability irrespective of *IL36R* KD treatment (Supplementary Figure 2). The above results suggest that LXR could play a role in the IL-36 dependent restriction of bacterial growth.

Since IL-36 can regulate the production of APs^7,11,12^ and since APs potently inhibit *Mtb* growth^29–34^, we next addressed the role of the IL-36/LXR axis in the regulation of such molecules. Of note, we identified several predicted binding sites for LXRα in the proximity of several AP sequences (Supplementary Table 1). Incubation of THP-1 macrophages or MDM with rIL-36γ and LXR agonists triggered the expression of the APs, cathelicidin (*CAMP*), β-defensin 1 (*DEFB1*) and β-defensin 2 (*DEFB4*), which could be blocked by LXR inhibitors (Fig. 3C,D). In contrast, LXR inhibition did not block vitamin D-induced expression of APs, showing that vitamin D triggers AP production independent of the IL-36/LXR axis (Fig. 3C,D). Importantly, similar results were observed upon *Mtb* infection, triggering the LXR- and IL-36-dependent induction of APs (Fig. 3E,F). These results could also be validated at the protein level (Fig. 3G). Our findings demonstrate that LXR activation is necessary for IL-36-induced AP production and inhibition of *Mtb* growth.

**Figure 4:**
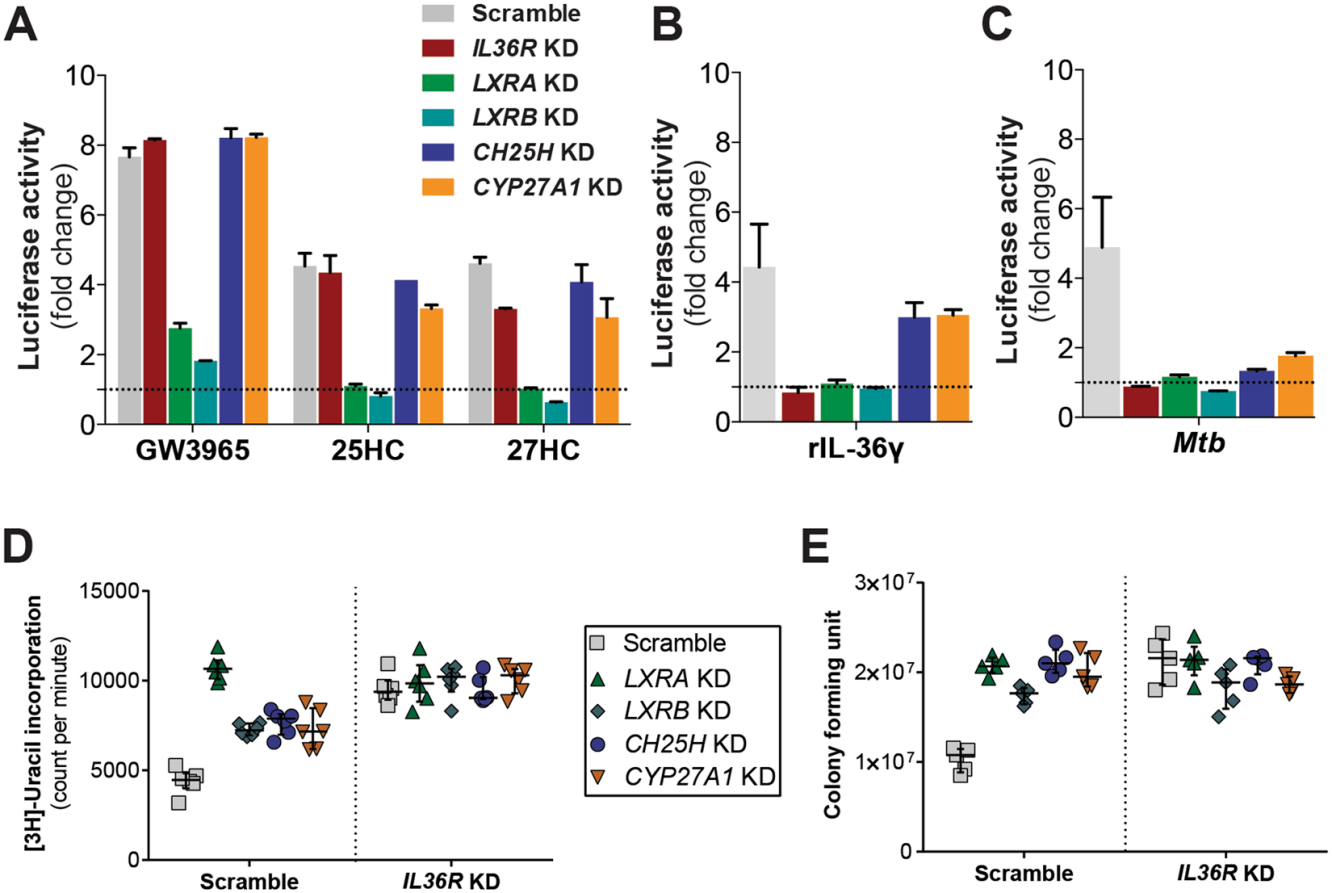
Deficient IL-36/LXR axis in macrophages allows *Mtb* growth. (**A**–**C**) LXR luciferase activity of scramble versus *IL36R*-, *LXRA*-, *LXRB*-, *CH25H*-, *CYP27A1*-deficient macrophages after 8 h cell stimulation with (**A**) 500 nM of GW3965, 25HC, or 27HC, (**B**) 25 ng/ml rIL-36γ or (**C**) after 15 h *Mtb* infection. (**D** and **E**) Bacterial growth assessed by [3 H]-uracil incorporation (**D**) and CFUs (**E**) in IL-36 signaling deficient dual knockdown macrophages. (**A–C**) Data from one representative experiment of two independent experiments are shown. Data are shown as mean ± SD of technical replicates. (**D** and **E**) Bacterial growth results are representative of one experiment of two independent experiments with at least five replicates each. Data are shown as median ± interquartile range.

We further dissected this IL-36/LXR pathway by generating gene specific KD in LXR luciferase reporter cells or secondary/dual KD in scramble control and *IL36R* KD cells. KD efficiency for genes *LXRA*, *LXRB*, *CH25H* and *CYP27A1* genes, encoding human LXRα, LXRβ, CH25H and the Cyp27a1 enzyme, were similar for all these three cell lines (Supplementary Figure 3A). Testing different LXR ligands (GW3965, 25HC and 27HC) in the various KD cells, we observed that both *LXRA* and *LXRB* are necessary for LXR activation induced by these ligands (Fig. 4A). Furthermore, as observed for *IL36R* KD cells, neither exogenous rIL-36γ nor *Mtb* infection were able to induce LXR dependent luciferase activity in the absence of *LXRA* and *LXRB* (Fig. 4B,C). In addition, we observed a significant reduction of LXR activity in *CH25H* and *CYP27A1* KD cells upon *Mtb* infection or IL-36 stimulation (Fig. 4B,C), indicating the involvement of these enzymes in the IL-36/LXR axis. Next, we examined the growth of *Mtb* in scramble and *IL36R* KD cells, with additional KD of *LXRA*, *LXRB*, *CH25H* or *CYP27A1*, using [3 H]-uracil uptake and CFU assay. As shown in Fig. 4D,E, control of bacterial growth was impaired in LXR deficient cells to a similar extent as observed in the scramble-transduced *IL36R* KD cells, while no significant difference in cell viability was detected (Supplementary Figure 3B). Inhibition of the cholesterol-converting enzymes CH25H or CYP27A1 leads to higher bacterial growth rates compared to scramble control. These findings suggest the requirement of LXRα, LXRβ, CH25H and CYP27A1 in IL-36 induced host defense against *Mtb* infection.

### IL-36 and LXR axis contribute to cholesterol metabolism

**Figure 5:**
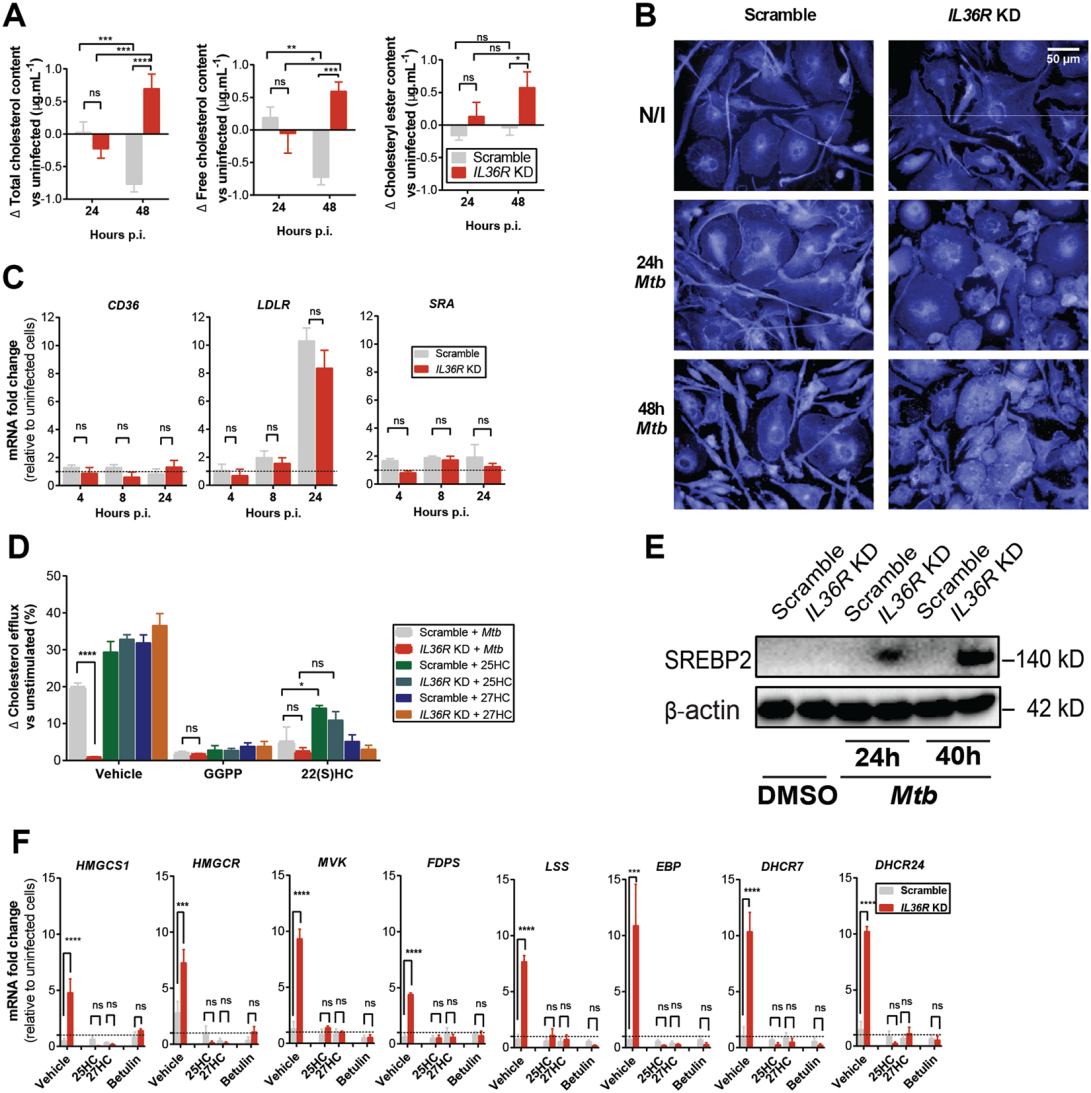
IL-36 signaling modulates cholesterol metabolism in *Mtb*-infected macrophage. (**A**) Changes in total cholesterol, free cholesterol and cholesteryl ester in cell lysates from scramble and *IL36R* KD THP-1 macrophages upon *Mtb* infection. (**B**) Immunofluorescence staining with Filipin III in non-infected (N/I), 24 h and 48 h *Mtb*-infected scramble versus *IL36R* KD THP-1 macrophages (40× objective magnification). (**C**) mRNA expression of *CD36*, *LDLR*, *SRA* in *Mtb*-infected scramble versus *IL36R* KD cells. (**D**) Cholesterol efflux upon *Mtb* infection or LXR stimulation in scramble versus *IL36R* KD THP-1 macrophages with or without LXR inhibitors. (**E**) SREBP2 protein expression in *Mtb*-infected scramble and IL-36 deficient cells. (**F**) mRNA expression of several cholesterol synthesis genes in *Mtb*-infected macrophages at 30 h with or without 27HC, 25HC and betulin stimulation. (**A**,**C**,**D**,**F**) Data pooled from three independent experiments are shown. Data are shown as mean ± SD. (**B** and **E**) Data from one representative experiment of at least two independent experiments are shown. *P* values: ns *p* > 0.05; *p ≤ 0.05; **p ≤ 0.01; ***p ≤ 0.001; ****p ≤ 0.0001.

The LXR pathway plays a central role in cholesterol homeostasis^16,18^. Since we have observed an IL-36-dependent LXR activation upon *Mtb* infection, we decided to evaluate the effect of IL-36 on cholesterol levels in infected cells. Total cholesterol levels were significantly elevated in IL-36 signaling deficient cells, whereas infection decreased cholesterol levels in control cells over time (Fig. 5A). Increased accumulation of free cholesterol in deficient cells was also observed by Filipin III fluorescence staining, revealing elevated numbers of cholesterol droplets in infected *IL36R* KD cells (Fig. 5B). Because of the known role of CD36, LDLR and SRA in cholesterol uptake^35,36^, we assessed their expression. No significant differences were observed at the mRNA level upon infection, when comparing IL36R proficient and deficient cells (Fig. 5C). Next, we evaluated cholesterol efflux by measuring cholesterol in supernatants, relative to the total cholesterol. As shown in Fig. 5D, elevated cholesterol efflux in response to *Mtb* infection was abrogated in IL-36 deficient cells. As expected, cholesterol efflux was significantly impaired in the presence of LXR inhibitors, whereas LXR ligands induced cholesterol efflux irrespective of IL-36 or *Mtb* infection (Fig. 5D).

In addition to measuring cholesterol efflux, we further studied the effect of *Mtb* infection on cholesterol synthesis by evaluating the expression of 24 genes related to cholesterol biosynthesis (Supplementary Figure 4)^18^. Consistent with the results in Fig. 5A, most genes were down regulated upon infection, whereas IL-36 deficient cells showed increased expression at later time points post infection (Supplementary Figure 4). The transcription of these genes is regulated by the transcription factor sterol regulatory element binding protein 2 (SREBP2)^37^, which also showed elevated expression in IL-36 deficient cells upon infection (Fig. 5E). These results point to an important role of IL-36 in the regulation of cholesterol biosynthesis. To determine the role of SREBP2 in this regulation, we used betulin, a specific inhibitor of the SREBP pathway independent of LXR activation^38^. In addition, we also used oxysterols (25HC and 27HC), all of which can act similarly as intracellular cholesterol suppressors^39^. As expected, the effects of betulin and oxysterols were comparable, suppressing the elevated expression of cholesterol biosynthesis genes upon *Mtb* infection in IL-36 deficient cells (Fig. 5F). Our results suggest that endogenous IL-36 signaling regulates cholesterol metabolism during *Mtb* infection through LXR activation, both by induction of cholesterol efflux and by suppression of its synthesis.

### Suppression of intracellular cholesterol synthesis reduces *Mtb* growth

**Figure 6:**
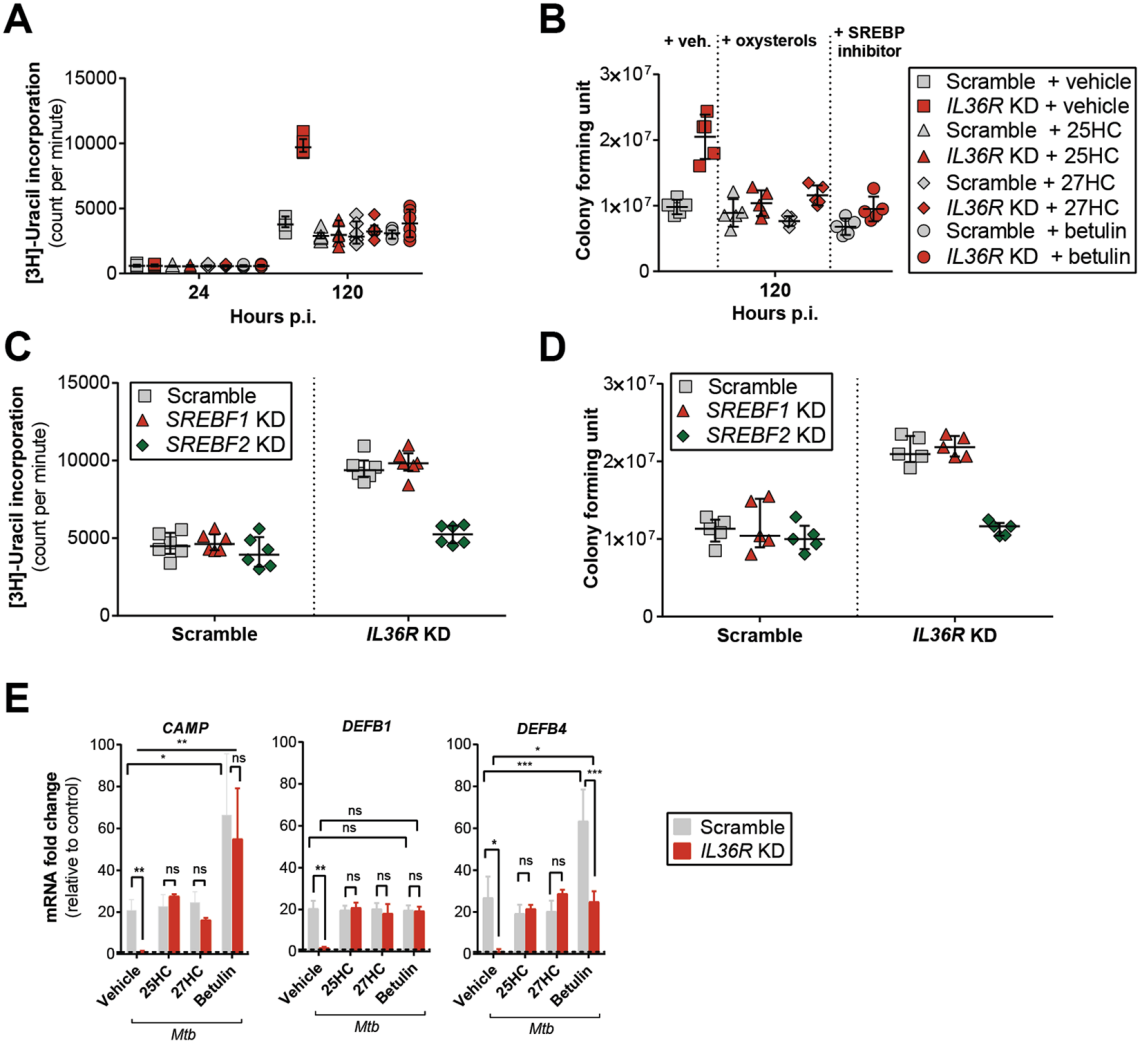
Oxysterols and inhibition of SREBP2 exhibit antibacterial effects. Quantification of (**A**) [3 H]-uracil uptake and (**B**) colony forming units (CFUs) of *Mtb*-infected scramble versus *IL36R* KD macrophages with simultaneous incubation of vehicle, 25HC, 27HC or betulin. (**C** and **D**) Bacterial viability in scramble, *SREBF1* KD and *SREBF2* KD macrophages at 120 h *Mtb* infection assessed by (**C**) [3 H]-uracil uptake assay and (**D**) CFU counts (**E**) mRNA expression levels of APs 24 h post *Mtb* infection of scramble and *IL36R* KD THP-1 macrophages, in the presence or absence of 25HC, 27HC or betulin. (**A** and **B**) Data from one representative experiment of three independent experiments are shown. (**C** and **D**) Data from one representative experiment of two independent experiments are shown, with at least five replicates each are shown. (**E**) Data pooled from three independent experiments are shown and presented as mean ± SD. *P* values: ns p > 0.05; *p ≤ 0.05; **p ≤ 0.01; ***p ≤ 0.001.

Since we observed differences in cholesterol metabolism, which depended on IL-36 signaling, we sought to determine whether suppression of cholesterol biosynthesis by natural LXR ligands or the SREBP inhibitor could affect bacterial growth. Interestingly, stimulation with all these compounds restored control of mycobacterial growth, which was lost in *IL36R* KD cells (Fig. 6A,B). As control, the tested compounds did not affect cell viability (Supplementary Figure 5A).

Moreover, since betulin can inhibit both SREBP1 and SREBP2^38^, we further evaluated the bacterial growth using scramble and *IL36R* KD cells with additional *SREBF1* and *SREBF2* gene KD (Supplementary Figure 5B), which encode SREBP1 and SREBP2, respectively^38^. Only *IL36R* KD cells lacking *SREBF2* were able to control bacterial growth under IL-36 signaling deficiency (Fig. 5C,D), and there was no difference in cell viability among these cells (Supplementary Figure 5C). These findings confirm the role of cholesterol homeostasis in macrophage-dependent host defense against *Mtb* infection.

Furthermore, following our previous results pointing to a host defense mechanism involving AP production, we extended our analysis to the previously mentioned APs. Consistent to previous results in Fig. 3, stimulation with the oxysterols (25HC and 27HC) induced expression of these APs in infected *IL36R* KD cells to levels seen in scramble cells (Fig. 5E). In sum, these results support the notion that LXR regulation of cholesterol metabolism plays a major role in IL-36-induced host defense to *Mtb* mediated by macrophages.

## Discussion

We have previously demonstrated that IL-36 production is triggered by *Mtb* infection, and that IL-36 plays a critical role in limiting bacterial growth^7^. While the previous study focused on the mechanism of IL-36 production, here we dissected the downstream effects. Using a human macrophage infection model, we describe a novel cellular regulatory mechanism connecting IL-36 signaling and cholesterol metabolism in host defense against *Mtb*. We demonstrate that in response to *Mtb* infection, IL-36 activates the LXR pathway, the expression of its natural ligands 25HC and 27HC, as well as the enzymes involved in cholesterol synthesis. In turn, activation of LXR regulates cellular cholesterol levels, predominantly by concomitant induction of cholesterol efflux and suppression of its biosynthesis. Moreover, we demonstrate that the IL-36/LXR axis is also involved in the production of APs, further strengthening the role of IL-36 in host defense against *Mtb*.

In lung phagocytes the LXR pathway, and its target genes, have been previously shown to play a major role in early host defenses to bacterial infections, including *Mtb*^14^. In macrophages, ABCA1 limits mycobacterial load^40^, whereas down-regulation of ABCG1 favors persistence of *Helicobacter cinaedi*^41^. Moreover, ApoE deficient mice have impaired immunity to *Mtb*^42^, *Listeria monocytogenes*^43^ and *Klebsiella pneumonia*^44^. In the present study, we demonstrate IL-36R dependent up-regulation of LXR target genes upon *Mtb* infection. We show that endogenous IL-36, induced upon *Mtb* infection^7^, signals through IL-36R and activates LXR. Strikingly, upon inhibition of LXR signaling, the bacterial burden was increased, emphasizing an important role for LXR in the control of infection. In contrast, this increase was not observed when LXR was inhibited in *IL36R* deficient cells, pointing to a crosstalk between IL-36 and LXR signaling pathways, and its role in infection control. Since we did not observe any differences in bacterial uptake in the IL-36R deficient cells, we consider it unlikely that phagocytosis was affected by IL-36 mediated host defense mechanisms.

LXR activation can be mediated by endogenous oxysterols such as 25HC and 27HC^24–26^, that are expressed in myeloid cells^25,45^. Here we show that *Mtb* infection, as well as endogenous IL-36γ^7^, stimulated expression of these oxysterols. Moreover, we confirmed that the induction of both, the oxysterols and the enzymes responsible for their conversion was dependent on IL-36 signaling. We cannot exclude the possibility that other oxysterols, induced in an IL-36 dependent manner upon *Mtb* infection can also trigger the LXR pathway. Indeed, we observed that the expression of *CYP46A1*, responsible for the production of 24(S)HC^27^, was also induced upon infection in an IL-36-dependent way. Surprisingly, the induction of 25HC and 27HC was not observed upon stimulation with IL-1β, which is also induced upon *Mtb* infection^9^, indicating distinct biological effects by different members of the IL-1 family. A reverse mechanism where these LXR agonists can also modulate cytokine expression in macrophages has been reported^46^. It was shown that oxysterols can induce or repress the expression of pro-inflammatory cytokines like IL-1β, IL-6 and IL-8, depending on the context^46–49^.

In the present study, we identified an IL-36/LXR dependent regulation of AP production upon *Mtb* infection. More precisely, we show that upon infection, the expression of several APs, such as cathelicidin and β-defensin2, is regulated by IL-36 and LXR dependent mechanisms. In agreement with these results, LXR stimulation by the synthetic ligand T0901317 has previously been shown to induce β-defensin 1 (*DEFB1*)^50^, and the LXR target gene *AIM* or *CD5L* was found to be involved in the induction of cathelicidin and β-defensin 2 upon mycobacterial infection^51^.

Taking into consideration the observed increased SREBP2 expression in *IL36R* deficient cells and the fact that the SREBP inhibitor betulin^38^ can boost the expression of these APs upon *Mtb* infection, it is tempting to assume that the IL-36 dependent antimicrobial responses are indeed mediated by this mechanism. Accordingly, we revealed that the effect of LXR activation by LXR agonists (25HC or 27HC) or SREBP inhibition (betulin) reverted the decreased control of *Mtb* replication observed in an IL-36 signaling deficient context (Fig. 6A and C). Moreover, upon KD of *SREBPF2* (the gene encoding SREBP2), but not *SREBF1* which encodes SREBP1, on *IL36R* deficient cells, we could reverse the increased bacterial replication to the levels compared to IL36R proficient cells (scramble, Fig. 6C,D). Altogether, these results point to an involvement of SREBP2 in the biological effects mediated by the IL-36 axis on *Mtb* replication. Cationic APs such as cathelicidin and defensins interact with the bacterial surface directly^52,53^ and serve as immune mediators to attract and activate immune cells during the innate immune response^53,54^. Our results favor a mechanism where in response to *Mtb*, IL-36 triggers AP production via LXR activation to control mycobacterial growth.

Suppression of cellular cholesterol levels has been implicated as a host defense mechanism against infectious agents^39,40^. In the present study we document an IL-36-dependent cholesterol reduction in *Mtb*-infected macrophages via the activation of LXR and SREBP pathways. Reduced cholesterol abundance likely affects the persistence of *Mtb*^21,27^ and its uptake by perturbing the composition of membrane cholesterol^39^. Others have reported an increased accumulation of intracellular lipids in the context of TB, both upon *in vitro Mtb* infection and in human granulomas^55–58^. Reprogramming of host lipids by *Mtb* in murine macrophages has been previously reported by *Ouimet et al*.^59^. This mechanism involves microRNA-33 (Mir-33), resulting in increased expression of srebf2 and downregulation of ABCA1^59,60^. However, species related differences, between mouse and human, in the role of Mir-33 in the regulation of cholesterol homeostasis have been documented^60^, and could account for the differential findings. In addition, it has also been shown that this accumulation is dependent on oxygen availability, elicited under hypoxic conditions^56^. Since we have performed all our studies under normoxia (21% O_2_), we cannot exclude the possibility that under different conditions, e.g. hypoxia such as in granulomas, other possible mechanisms and respective phenotypes exist.

In sum, we have identified a novel IL-36-LXR-cholesterol axis involved in macrophage-mediated host defense against *Mtb* infection. Further studies are required to verify whether modulation of this axis by exogenous natural LXR ligands or the SREBP inhibitor or similar compounds can be harnessed for host-directed therapy against tuberculosis. *In vivo* validation studies using human samples and *IL36R* KO mice will further help to decipher the relevance of the IL-36-LXR cholesterol axis in tuberculosis control.

## Methods

### Antibodies and reagents

The following unconjugated antibodies (Abs) were used for immunoblotting: rabbit anti-ABCA1, rabbit anti-LXRα, rabbit anti-LXRβ from Sigma-Aldrich (Saint Louis, Missouri, USA); chicken anti-ABCG1 from Thermo Fischer Scientific (Darmstadt, Germany), rabbit anti-SREBP2 from Bethyl Laboratories/Biomol (Montgomery, Texas, USA); goat anti-hCAP18/LL37, goat anti-hBD1 and rabbit anti-hBD2 from Santa Cruz Biotechnology (Heidelberg, Germany). The following HRP-conjugated Abs were used: mouse anti-beta actin from Sigma-Aldrich; anti-rabbit IgG from Cell Signaling (Leiden, The Netherlands); anti-goat IgG from R&D Systems (Minneapolis, Minnesota, USA) and anti-chicken IgY HRP conjugated from Thermo Fischer Scientific.

Human recombinant IL-36α (rIL-36α) and GW3965 were purchased from Biozol Diagnostica (Eching, Germany). Human rIL-36β, rIL-36γ, rIL-36Ra, rIL-1β and granulocyte-macrophage colony stimulating factor (GM-CSF) were obtained from Peprotech (Hamburg, Germany). Dimethyl sulfoxide (DMSO), 27-hydroxycholesterol (27HC) and geranylgeranyl pyrophosphate (GGPP) were supplied from Enzo Life Sciences (Loerrach, Germany). 22(S)-hydroxycholesterol (22(S)HC) was obtained from Sigma-Aldrich. 25-hydroxycholesterol (25HC) and the active form of vitamin D, 1,25-dihydroxy vitamin D_3_ (1,25(OH)_2_D_3_), were purchased from Biomol (Hamburg, Germany). PMA (Phorbol 12-myristate 13-acetate) and betulin were purchased from Calbiochem (Darmstadt, Germany).

### Cell culture and macrophage differentiation

THP-1 (ATCC: TIB 202) human monocytic cell line-derived macrophages (THP-1 macrophages) and primary human monocyte-derived macrophages (MDM) were cultured in RPMI 1640 (Gibco, Darmstadt, Germany). HEK293T (human embryonic kidney epithelial cells, ATCC: CRL-11268) were cultured in DMEM. Both culture media were supplemented with 10% (v/v) heat-inactivated FBS (Gibco), 1% (v/v) sodium pyruvate (Gibco), 1% (v/v) penicillin-streptomycin (Gibco), 1% (v/v) L-glutamine, 1% (v/v) HEPES buffer (Gibco), 0.05 M 2-mercaptoethanol (Gibco). THP-1 monocytes (ATCC: TIB-202) were differentiated into macrophages using 100 nM PMA. For the generation of human MDM, peripheral blood CD14^+^ cells were positively selected by MACS using CD14^+^ cell isolation kit (Miltenyi Biotech, Bergisch Gladbach, Germany) according to manufacturer’s instructions and differentiated with GM-CSF at 20 ng/ml for 7 days in RPMI 1640. Cells were kept at 37 °C in 5% CO_2_. Buffy coats were provided by the German Red Cross (*Deutsches Rotes Kreuz*; ethical registration no. EA1/353/14). All experiments without infection were performed under biosafety level (BSL) 2 conditions.

### Lentivirus production

Lentiviruses were produced according to the described TRC lentiviral proceedings (https://www.broadinstitute.org/genome_bio/trc/publicProtocols.html). Briefly, HEK293T cells were seeded at a density of 2 × 10^5^/ml in DMEM in 96 well plates. After 24 h of incubation, cells were transfected with lentiviral packaging mix (Sigma-Aldrich) and 100 ng ng of the shRNA (listed in Supplementary Table 2) containing pLKO.1-puro vector (Sigma Aldrich), using Fugene 6 (Roche, Berlin, Germany) in Optimem medium (Gibco). After 18 h of incubation, medium was replaced with high serum growth medium (30% FCS (v/v)). Viruses were harvested at 48 h and 72 h post-transfection.

### Lentiviral transduction

Lentivirus-based transduction of shRNA (Supplementary Table 2) and LXR luciferase reporter was performed based on the protocol at the RNAi Consortium website (https://www.broadinstitute.org/genome_bio/trc/publicProtocols.html). Briefly, THP-1 cells were resuspended in medium containing 8 μg/ml polybrene (Sigma-Aldrich) at a density of 1 × 10^6^ cells/ml. Cell suspension was added to plates containing virus and centrifuged for 90 min at 2200 rpm at 37 °C. Stable knockdown (KD) and LXR reporter cell lines were further selected using 5 μg/ml puromycin. For KD efficiency, each clone was grown for 2 weeks and gene expression evaluated. The clone with the highest KD efficiency was expanded for further studies. For LXR reporter generation, cells were selected and grown from single cell sorted clones and further tested with specific LXR ligand, GW3965^61^.

### Microarray Data Analysis

Gene expression profiles were generated from *Mtb*-infected IL-36R KD and scramble macrophages from three independent experiments. RNA was extracted using Trizol (Life Technologies, Ober-Olm, Germany), labeled and hybridized to Agilent whole-genome 4 × 44 k human expression arrays. Raw data were analyzed using the R package Limma. Data were log-transformed, and differentially expressed genes were identified based on p-value < 0.01 after Benjamini-Hochberg correction for multiple testing. To identify enriched signaling pathways, differentially expressed genes were analyzed using Ingenuity Pathway Analysis (IPA) (Ingenuity Systems, Redwood City, USA). The microarray data are deposited in https://www.ncbi.nlm.nih.gov/geo/ with database entry: GSE103092.

### LXR luciferase assay

Cells containing LXR luciferase reporter were seeded at a density of 2 × 10^4^ cells/well in 96 well plates. Following desired stimulations, cells were lysed using 30 μl of 1 × Reporter Lysis Buffer (Promega, Mannheim, Germany) and used to determine luciferase activity using Dual-Glo Luciferase Assay System (Promega) according to the manufacturer’s instructions. Luciferase activity was normalized to the amount of protein determined by BCA Protein Assay kit (Thermo Fischer Scientific). Results are shown as fold change of luciferase activity determined by normalizing luminescence values relative to control cells at equal protein quantity. Non-stimulated control cells were exposed to equal concentrations of DMSO (0.01–0.1%) or vehicle (a mix of DMSO and ethanol 0.01–0.1%) in culture medium depending on the solvent of stimulants, oxysterols or inhibitors.

### Mycobacterial culture and *in vitro* infection

*Mtb* strain H37Rv (ATCC: 27294) was grown to an early log phase under BSL 3 conditions in Middlebrook 7H9 broth (BD, Heidelberg, Germany) supplemented with 0.05% glycerol and Tween-80, and 10% ADC enrichment (BD). For *in vitro* infection, bacteria were maintained in log growth phase at OD600 nm between 0.2–0.6. Bacteria were centrifuged and resuspended with PBS. Single bacteria were obtained by passing through a syringe and resuspended in culture media at desired concentrations. An OD of 1 is equivalent to 2 × 10^8^ bacteria. All infections were done continuously at a multiplicity of infection (MOI) of 10 under BSL 3 conditions.

### RNA extraction and qPCR

RNA was isolated using Trizol in experiments with *Mtb* infections. In experiments without *Mtb* infection, RNAeasy Plus kit (Qiagen, Hilden, Germany) was used for RNA extraction. After quantification of RNA concentration using a Nanodrop 2000c (Thermo Fischer Scientific), RNA was reverse-transcribed at equal concentration using iScript cDNA Sythesis Kit (Bio-Rad, Munich, Germany) and then proceeded for real time quantitative PCR analysis using Power SYBR Green (Applied Biosystem, Darmstadt, Germany) in a LightCycler480 thermocycler (Roche). Primers are listed in Supplementary Table 3. *B2M* was used as internal control. All gene expression data were normalized to the average of uninfected/vehicle or DMSO-stimulated samples. Fold change is the result of normalized Ct value assuming delta Ct = 1 equals a fold change of 2.

### Immunoblotting

Cells were lysed in ice-cold 1× RIPA buffer supplemented with a cocktail of protease inhibitors (Santa Cruz Biotechnology), and centrifuged for 20 min in filter SPIN-X tubes (Sigma-Aldrich). Soluble proteins were diluted at equal concentrations in 2× Laemmli buffer and denatured at 95 °C for 5 min. Cell lysates were separated by 4–15% Mini Protean SDS PAGE gel (Bio-Rad) and transferred onto nitrocellulose membrane (Roche). Blots were incubated with the specified antibodies. Beta actin was used as loading control at 42 kD band. For band visualization, blot membranes were incubated with SuperSignal West Pico Chemiluminescent Substrate (Thermo Fischer Scientific) and documented using ChemiDoc XRS+ Imaging System and Lab Imaging software (Bio-Rad).

### ELISA

Cell lysates were isolated from infected or stimulated cells in ice-cold Cell Lysis Buffer (Sigma-Aldrich) and mixed thoroughly. Samples from infected cells were transferred and filtered through SPIN-X tube (Sigma-Aldrich) prior to assay. Samples were further diluted in PBS (1:5) and assayed for oxysterol quantities using the kit as following: 25HC competitive ELISA kit from Biozol Diagnostica and 27HC sandwich ELISA kit from Abbexa (supplied by Hoelzel Diagnostica, Cologne, Germany). All procedures were performed according to manufacturer’s instructions.

### Bacterial growth quantification

Colony forming units (CFUs) and [3 H]-uracil uptake were measured in parallel for bacterial growth at the specified time points. For CFUs, cells were lysed in 0.1% Triton-X in dH20 and serially diluted. Bacteria were plated onto 7H11 Middlebrook agar, sealed with paraffin film, and incubated for 21 days at 39 °C. Bacterial growth and growth inhibition were determined by [3 H]-uracil incorporation as previously described^62^.

### Cell-based cholesterol quantification

Cells were seeded at 1 × 10^6^ cells/well in 6 well plates and further infected or kept untreated for 24 h and 48 h. Cells were collected using cell scrapers (Thermo Fischer Scientific) and pelleted in two different tubes per sample. First half of pellets were extracted in 200 μl Chloroform:Isopropanol:NP-40 (Life Technologies) at ratio 7:10:0,1 and subsequently processed using Cholesterol/ Cholesteryl Ester Quantitation Kit (Calbiochem) according to the manufacturer’s instruction. The second half of pellets was assayed for protein concentration using Pierce BCA Protein Assay kit (Thermo Fischer Scientific). All values containing the quantity of total cholesterol, free cholesterol and cholesterol ester were normalized to total protein quantity and subtracted by the background values from uninfected cells.

### Filipin III free cholesterol staining

Cells were seeded at density of 2 × 10^5^ cells/well on sterile coverslips in 24 well plates. Cells were then infected with *Mtb* or left uninfected and fixed at 24 h and 48 h with 4% paraformaldehyde (Electron Microscopy Sciences, Munich, Germany) for 1 h and then washed with PBS three times. To stain with Filipin III (Biomol), stock solution was diluted to 1:100 with PBS/BSA 3% and incubated for 2 h at RT in the dark. Cells were then visualized using Leica DMR microscope (Leica Microsystems, Wetzlar, Germany) under UV light filter set (340–380 nm) and captured at 40× magnification.

### Cholesterol efflux assay

To determine cholesterol efflux, cells were plated at 2 × 10^4^ cells/well in 96 well plates (100 μl/ml) and labeled with fluorescent-labeled cholesterol for 16 h using reagents in Cholesterol Efflux Assay Kit (Sigma-Aldrich) according to the manufacturer’s protocol. In parallel, incubation with GGPP and 22(S)HC was performed for 15 h and 3 h respectively before washing procedure. Cells were treated or infected as required. After 24 h of incubation, supernatants and cell lysates were transferred into black well plates for fluorescence measurement at λex/λem: 482/515 nm in Infinite 200 PRO multimode reader (Tecan, Maennedorf, Switzerland). Each sample was also assayed for protein quantity using Pierce BCA Protein Assay kit (Thermo Fischer Scientific) and measured at an absorbance of 562 nm. Efflux was calculated as percentage of fluorescence intensity of medium divided by total fluorescence intensity of medium and cell lysate (normalized to protein quantity). The values obtained from unstimulated or uninfected cells were subtracted.

### Cell viability MTS assay

Cell viability was measured using colorimetric assay based on the reduction of MTS (3-(4,5-dimethylthiazol-2-yl)-5-(3-carboxymethoxyphenyl)-2-(4-sulfophenyl)-2H-tetrazolium) into formazan in supernatants using Celltiter96 Aqueous One Solution (Promega) according to manufacturer’s instructions.

### Statistical analysis

GraphPad Prism version 5 was used for statistical analyses. Student’s t-test was used for single comparison and one-way ANOVA test was performed for multiple comparisons followed by Bonferroni’s post-test. Data are shown as mean ± SD. Data derived from MDM samples, [3 H]-Uracil uptake assay and CFU counts were compared using Mann–Whitney test and are shown as median ± interquartile range. All data in this study are derived from at least three independent experiments, unless otherwise noted. Each independent experiment consisted of at least three technical replicates. All mean or median values calculated from each independent experiments were pooled for analysis as mentioned in each figure legend. *P* values were assigned as ns *p* > 0.05; *p ≤ 0.05; **p ≤ 0.01; ***p ≤ 0.001; ****p ≤ 0.0001.

## Electronic supplementary material

Supplementary Information
