# Supplementary material for: IL-36/LXR axis modulates cholesterol metabolism and immune defense to Mycobacterium tuberculosis
Source: Sci Rep. 2018 Jan 24;8:1520. doi: 10.1038/s41598-018-19476-x (PMC5784124; doi:10.1038/s41598-018-19476-x)
Supplement: Supplementary file 1 — Supplementary Information [file 41598_2018_19476_MOESM1_ESM.pdf]

Supplementary Information

IL-36/LXR axis modulates cholesterol metabolism and immune defense to *Mycobacterium tuberculosis*

Fadhil Ahsan, Jeroen Maertzdorf, Ute Guhlich-Bornhof, Stefan H.E. Kaufmann*, Pedro Moura-Alves*

Department of Immunology, Max Planck Institute for Infection Biology, Charitéplatz 1, Berlin Germany 10117

* Co-corresponding author

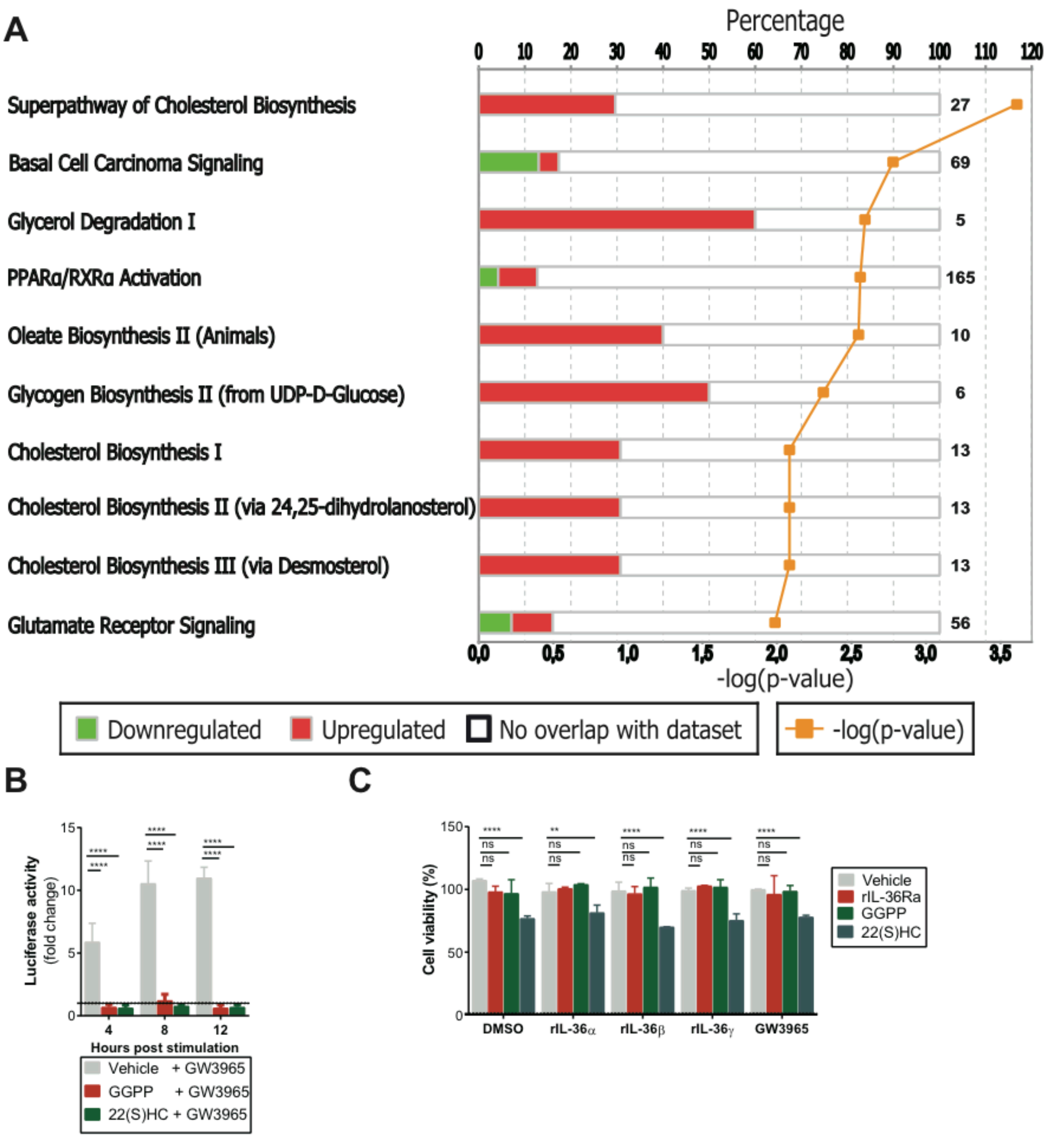

Supplementary Figure 1- IL-36-induced cholesterol synthesis regulation and LXR activation.

(A) Top 10 canonical pathways differentially regulated after Ingenuity Pathway Analysis (IPA) of differentially expressed genes in *Mtb*-infected IL-36R KD cells compared to scramble macrophages. Yellow line corresponds to the significance of gene enrichment shown in -log *P* value (Fisher's exact test) and the percentage of genes in each canonical pathway is distributed according to their regulation i.e. down (green bar), up (red bar) and no overlap with dataset (open bar). The total number of genes from each pathway is shown on top of each bar. **(B)** LXR luciferase reporter activity in GW3965 (500 nM)-stimulated THP-1 macrophages at 4, 8, 12 h, in the presence of absence of vehicle, GGPP and 22(S)HC for 15h and 3h, respectively. **(C)** Cell viability of THP-1 macrophages stimulated with DMSO, 25 ng/ml of rIL-36α, rIL-36β, rIL-36γ and GW3965 and previously incubated with vehicle, rIL-36Ra, GGPP and 22(S)HC. Data pooled from three independent experiments are shown. Data are shown as mean ± SD. *P* values shown as ns p>0.05; ** *p*≤0.01; **** *p*≤0.0001. Abbreviations: rIL-36Ra, recombinant IL-36 receptor antagonist, GGPP,

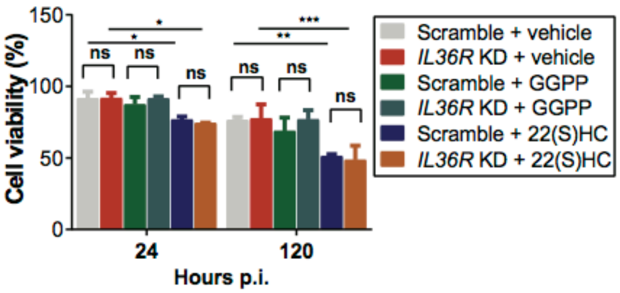

Supplementary Figure 2- Cell viability of macrophages upon *Mtb* infection. Cell viability of *Mtb*-infected scramble and *IL36R* KD THP-1 macrophages using MTS assay upon previous incubation with vehicle, GGPP or 22(S)HC. Data pooled from three independent experiments are shown. Data are shown as mean ± SD**.** *P* values shown as ns $p>0.05$; * $p\leq0.05$; ** $p\leq0.01$; *** $p\leq0.001$.

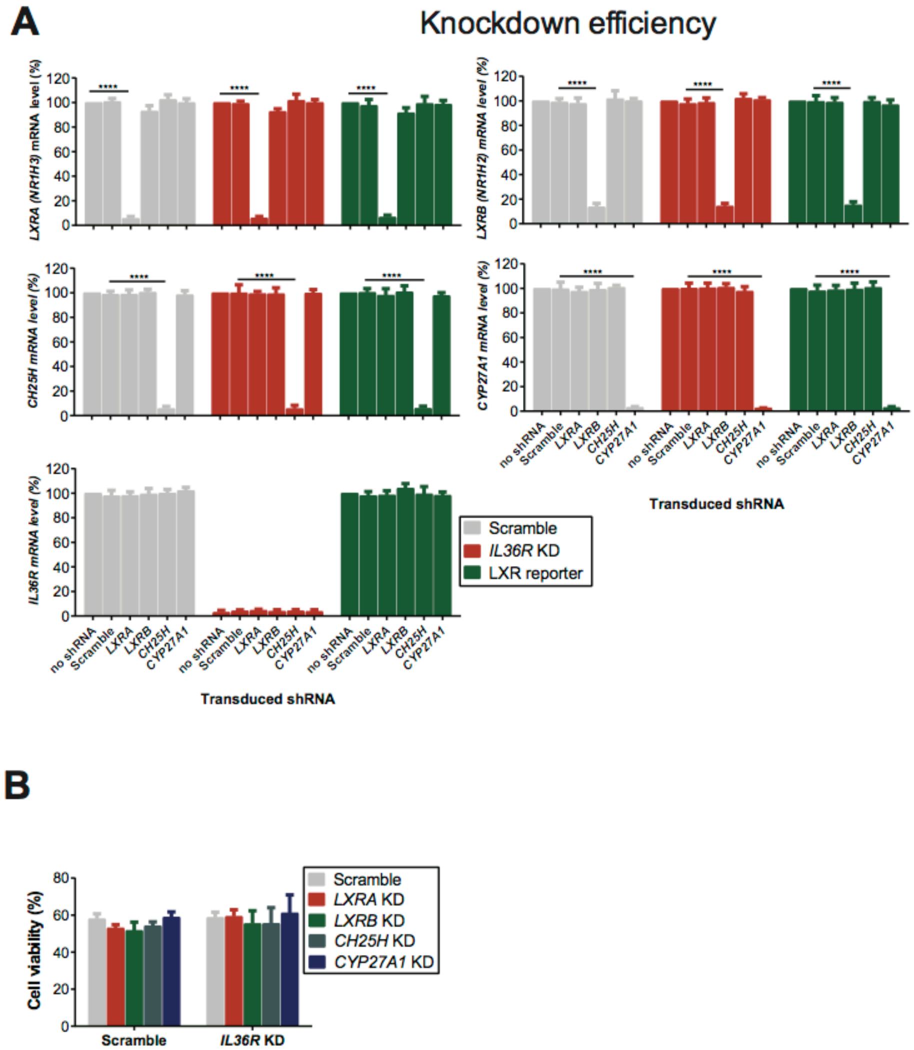

Supplementary Figure 3-KD efficiency of THP-1 cells and cell viability of *Mtb*-infected macrophages. **(A)** Scramble, *IL36R* KD and LXR reporter THP-1 cells were analyzed for KD efficiency post-secondary shRNA lentiviral transduction. **(B)** Cell viability post 120 h *Mtb* infection in scramble versus *IL36R* KD macrophages with secondary scramble or shRNA KD for *LXRA*, *LXRB*, *CH25H* and *CYP27A1*. Data pooled from at least two independent experiments are shown. Data are shown as mean ± SD. *P* values:

**** *p*≤0.0001.

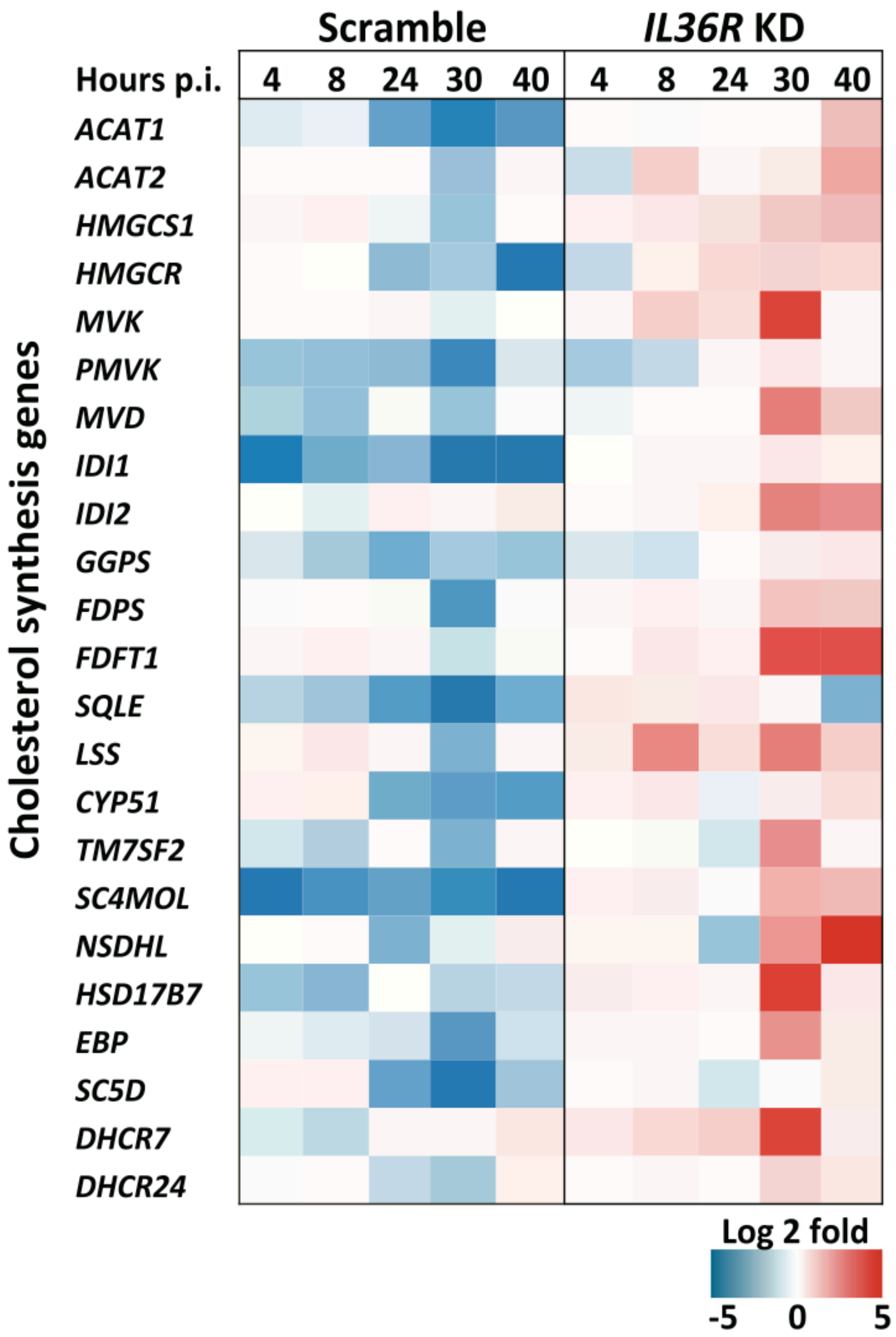

Supplementary Figure 4- IL-36 signaling modulates cholesterol metabolism in *Mtb*-infected macrophages. Heat map of mRNA expression levels for 24 cholesterol synthesis genes over time in scramble and *IL36R* KD macrophages. Expression levels are relative to uninfected cells. Upregulation and downregulation are shown in red and blue, respectively.

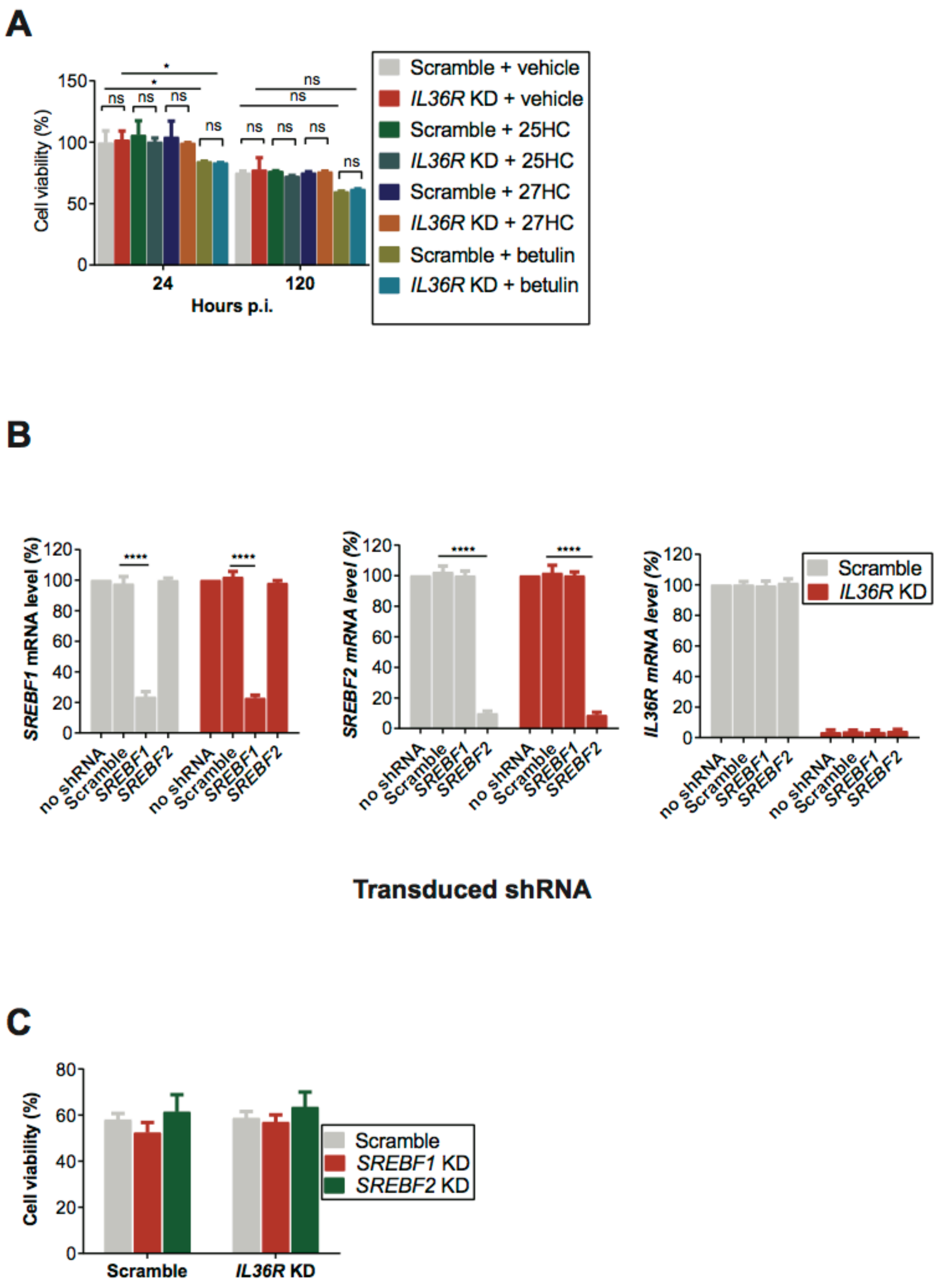

Supplementary Figure 5- Cell viability of *Mtb*-infected cells and KD efficiency. (A) Cell viability of infected scramble versus *IL36R* KD THP-1 macrophages incubated with vehicle, 25HC, 27HC and betulin at the time of infection. **(B)** KD efficiency after secondary shRNA lentiviral transduction of scramble and *IL36R* KD THP-1 cells. **(C)** Cell viability of scramble, *SREBF1* KD and *SREBF2* KD THP-1 macrophages post 120 h *Mtb* infection. Data pooled from three independent experiments are shown. Data are shown as mean ± SD. *P* values: ns p>0.05; * *p*≤0.05; **** *p*≤0.0001.

Supplementary Table 1- Predicted LXR related Transcription Factors of antimicrobial peptide genes*

Gene	Relative nucleotide distance of binding sites (strand)	Sequence motif	Predicted transcription factor (TF) : aligned TF
CAMP	6098 (+)<br>8567 (+) 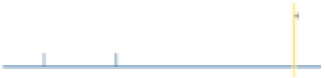	tgaccttaggTGATCca<br>tgacctcagaTGATCca	NR-DR (nuclear receptor direct repeat) : LXRα/β
DEFB1	9702 (-) 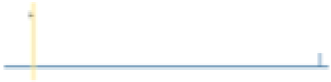	ctGGTCActagaggtca	NR-DR (nuclear receptor direct repeat) : LXRα/β
DEFB4	1374 (+)<br>9261 (-) 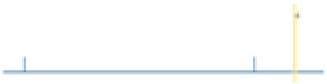	tgacctcatgTGATCca<br>cgGATCAcctgaggtca	NR-DR (nuclear receptor direct repeat) : LXRα/β

*The analysis was performed using TRANSFAC database through GeneXplain platform.

Supplementary Table 2- List of shRNA target sequences

**Gene**	**Target sequence (5’→3’)**
CH25H	AGAGAAACACCTGAGCTATAT
CYP27A1	ACTTTGCCTTGGAAGCTATTT
IL36R	GCCAGAGTCAATTCAGTACAT
LXRA	GTGCAGGAGATAGTTGACTTT
LXRB	GAAGGCATCCACTATCGAGAT
SCRAMBLE	CAACAAGATGAAGAGCACCAA
SREBF1	CCCTGTGCTGACGGAAGCCAA
SREBF2	CCTCAGATCATCAAGACAGAT

Supplementary Table 3- List of human primer sequences used in this study

**Gene**	**Forward sequence (5'→3')**	**Reverse sequence (5'→3')**
ABCA1	ACATCCTGAAGCCAATCCTGA	CTCCTGTCGCATGTCACTCC
ABCG1	GGGGTCGCTCCATCATTTG	TTCCCCGGTACACACATTGTC
ACAT1	TACCAGAAGTAAAGCAGCATGG	TCATTCAGTGTACTGGCATTGG
ACAT2	CCCAGCCAATGCTTCAGGAAT	AAGCCCACGTTTATCAGCTTC
APOE	GTTGCTGGTCACATTCCTGG	GCAGGTAATCCCAAAAGCGAC
B2M	GAGGCTATCCAGCGTACTCCA	CGGCAGGCATACTCATCTTTT
CAMP	GGCTGGTGAAGCGGTGTAT	TGGGTACAAGATTCCGCAAAAA
CD36	AAGCCAGGTATTGCAGTTCTTT	GCATTTGCTGATGTCTAGCACA
CH25H	CTCTACCAGCATGTGATGTTTGT	CATGTCGAAGAGTAGCAGGCA
CYP27A1	GGTGCTTTACAAGGCCAAGTA	TCCCGGTGCTCCTTCCATAG
CYP46A1	TCGTCACGAGTCCTGAGTC	GCGCACGGTACATCTTGGA
CYP7A1	GCAATTTGGTGCCAATCCTCT	GCACAACACCTTATGGTATGACA
CYPS1	ATAACCCAGCATCAGGGGAAA	CACAGTGGGAAAGTATCCATCAA
DEFB4	GGTGGTATAGGCGATCCTGTT	AGGGCAAAAGACTGGATGACA
DHCR24	CACTGTCTCACTACGTGTCGG	CCAGCCAATGGAGGTCAGC
DHCR7	GCAGGGGTTGTGAACAAGTAT	GAGACGGCATAGCCAAGGAT
EBP	CACAGGGGTCTTAGTCGTGAC	CCAGGTGAATGAACCCACACA
FDFT1	GCAACGCAGTGTGCATATTTT	CGCCAGTCTGGTTGGTAAAGG
FDPS	TGTGACCGGCAAAATTGGC	GCCCGTTGCAGACACTGAA
GGPS	TGGATTAGCAGTAGGTCTCATGC	CCCAAGTGTATTAAGTAGCGGTT
HBD1	ATGAGAACTTCCTACCTTCTGCT	TCTGTAACAGGTGCCTTGAATTT
HMGCS1	CTCTTGGGATGGACGGTATGC	GCTCCAACTCCACCTGTAGG
HMGCR	TGATTGACCTTTCCAGAGCAAG	CTAAAATTGCCATTCCACGAGC
HSD17B7	ATCTGGACATCATCTCGCAGT	AAGAGCTGTAGGGTTCCTTGC
IDI1	AACACTAACCACCTCGACAAGC	AGACACTAAAAGCTCGATGCAA
IDI2	AAGAGGAATTGCCATCTGAACG	CTTGGTGTTAAACAAGACAACGC
IDOL	AAACCTGAGAAACCGGATCTCC	GCTCCACGAAGAACTTGACTCTA
IL36R	TCCCGAAGAGTTGTGTTTTGG	TGAGTGTGTCAGTATGGCTTGA
LDLR	ACCAACGAATGCTTGGACAAC	ACAGGCACTCGTAGCCGAT
LSS	GCACTGGACGGGTGATTATGG	TCTCTTCTCTGTATCCGGCTG
LXRA	ACACCTACATGCGTCGCAAG	GACGAGCTTCTCGATCATGCC
LXRB	AGAACTAATGATCCAGCAGTTGG	TTGCTTAGCGAAGTCCACGAT
MVD	GGACCGGATTTGGCTGAATG	CCCATCCCGTGAGTTCCTC
MVK	GGAGCAAGGTGATGTCACAAC	CGGCAGATGGACAGGTATAAGT
NSDHL	AAGAGATGCACAGTGATCGGT	GCACCTGGGGATTATCAAACC
PMVK	CCTTTCGGAAGGACATGATCC	TCTCCGTGTGTCACTCACCA
SC4MOL	TGCTTTGGTTGTGCAGTCATT	GGATGTGCATATTCAGCTTCCA
SC5D	CTTCTGTGCAACACTGAGCTA	TCTCTCGACGGACTTGATTCTTT
SQLE	TGACAATTCTCATCTGAGGTCCA	CAGGGATACCCTTTAGCAGTTTT
SRA	CCAGGTCCAATAGGTCCTCC	CTGGCCTTCCGGCATATCC
SREBF2	AACGGTCATTCACCCAGGTC	GGCTGAAGAATAGGAGTTGCC
TM7SF2	AACTCAGGCAATCCGATTTACG	GGGTCGCAGTTCACAGAAATA
